# Arctic & Antarctic dermatology: a narrative review of cutaneous conditions of polar explorers and researchers

**DOI:** 10.1007/s00403-025-04182-2

**Published:** 2025-04-01

**Authors:** Ryan Scheinkman, Rahul Aggarwal, Rachel Summers, Garrett Kraft, Rachel Papavasilopoulos, Daniel Bister, Julia Sarama, Keyvan Nouri

**Affiliations:** https://ror.org/02dgjyy92 grid.26790.3a 0000 0004 1936 8606 Dr. Phillip Frost Department of Dermatology and Cutaneous Surgery, University of Miami Miller School of Medicine, Miami, FL USA

**Keywords:** Dermatology, Wilderness medicine, UV, Exposure, Xerosis

## Abstract

The intersection of historical, environmental, and modern research contexts creates a unique framework for understanding dermatological conditions in polar regions. The poles represent unique challenges to dermatological health ranging from difficulties in delivering healthcare in the extreme environmental characteristics of the poles. A narrative review of PubMed and Google Scholar between December of 2024 to January 2025 was performed analyzing sources relevant to dermatological environmental insults and healthcare delivery challenges at the Earth’s poles. Polar explorers are likely to be at anincreased risk of developing skin cancer because of the high albedo of snow and ice coupled with a thinner region of the ozone layer in the poles, increased risk of developing frostbite and could induced dermatoses, face barriers to obtaining dermatologic care, and face dermatological challenges with nutrient and vitamin deficiencies. As polar research grows, dermatologists can serve a crucial role by developing protocols for managing dermatologic complications, training healthcare personnel prior to polar missions, and providing remote mission support through telemedicine.

## Introduction

Many notable expeditions to explore Earth’s polar regions have shaped our understanding of human physiology and resilience in the context of extreme environments. The disastrous Franklin Expedition in 1845, that sought to discover the Northwest Passage and ended in the deaths of the entire crews of both the HMS Terror and HMS Erebus, demonstrated the hazard behind polar exploration and the threats posed by hypothermia, infection, and general physical and mental decline on polar explorers’ health [1, 2]. Robert Peary’s controversial claimed conquest of the North Pole in 1909 further highlighted humanity’s determination to reach these geographic extremes, while the ratification of the Antarctic Treaty in 1959 transformed the southern continent into an unprecedented international sanctuary for scientific research [3, 4].

The unique environmental conditions at Earth’s poles present distinct challenges to human skin health and function. This region is subjected to extremely cold temperatures (-60 °C average austral winter temperature at South Pole Station), especially in winter, while wind speeds can exceed 200 mph in Antarctic katabatic storms [5, 6]. Paradoxically, despite the frigid temperatures, these polar environments are essentially cold deserts, with extremely low humidity and precipitation [7]. During summer months, the phenomenon of midnight sun creates continuous daylight, while winter brings total darkness, contributing to both physiological and psychological stress on human inhabitants [8]. The poles also have thinner ozone layers, resulting in higher risks for exposures of ultraviolet (UV) exposure [9].

Today, the poles serve as vital research laboratories where scientists study everything from climate change and glaciology to astronomy and particle physics. Modern polar research stations, such as McMurdo Station in Antarctica and the Svalbard Research Park in the Arctic, house researchers year-round [10, 11]. Perhaps most challenging are the “overwinterers”– scientists and support staff who remain at these remote stations during the polar winter, when evacuation becomes nearly impossible due to extreme weather conditions [12]. These individuals face not only the physical challenges of polar environments but also the psychological impacts of isolation and darkness, both of which can manifest in cutaneous conditions.

**Fig. 1:**
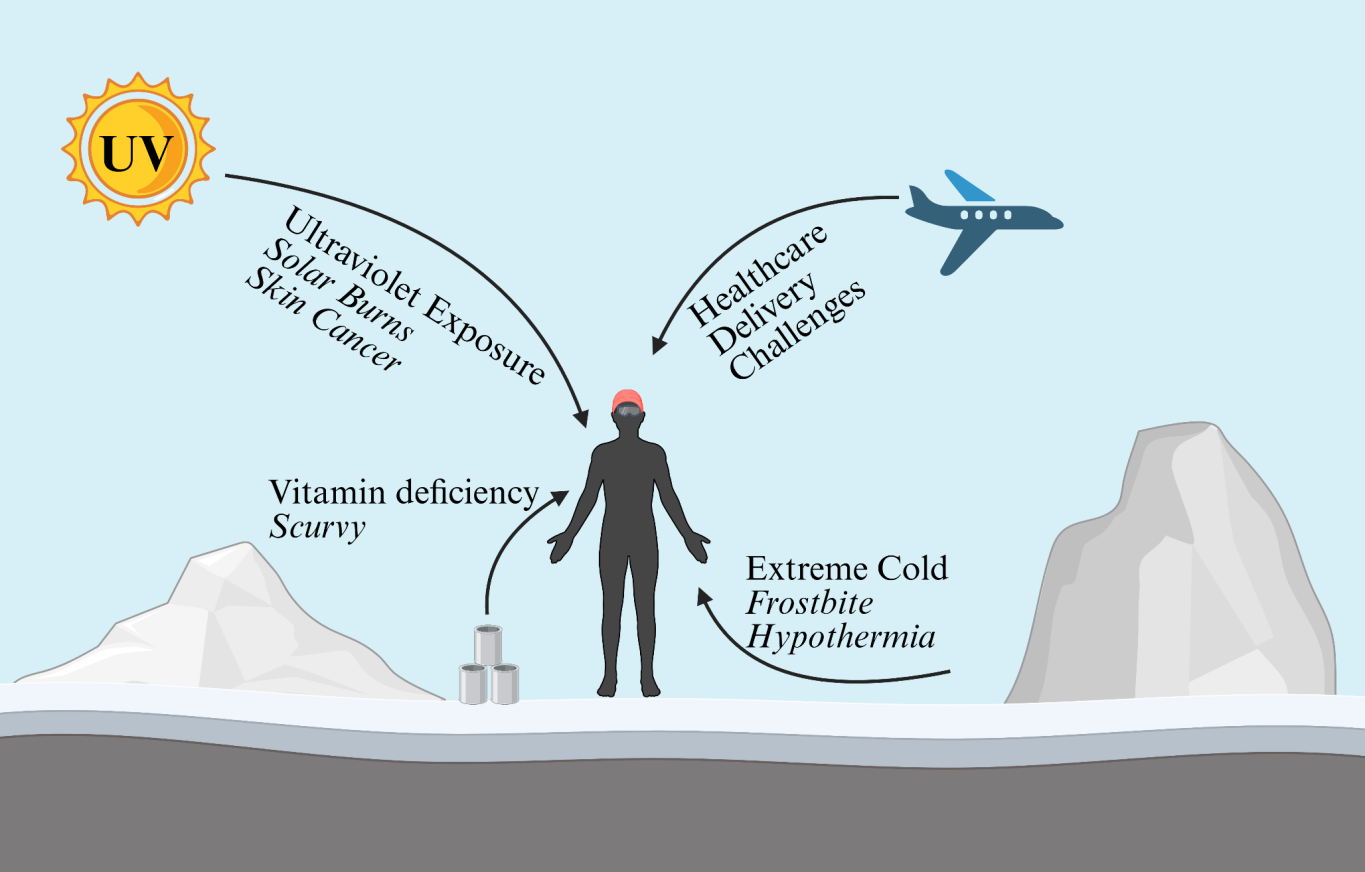
...

The intersection of these historical, environmental, and modern research contexts creates a unique framework for understanding dermatological conditions in polar regions. Since the beginning of polar exploration, explorers have faced dermatological diseases attributed to the environment and restrictions of their missions. For example, Franklin’s sailors were found to be suffering from scurvy (presents with adverse wound healing, ecchymoses follicular hyperkeratosis, perifollicular hemorrhages, edema) and lead poisoning (presents with Burton’s lines) [1, 2, 13, 14]. Contemporary explorers are at elevated risk for exposure, ultraviolet radiation-induced dermatoses, equipment-induced dermatoses, and other conditions caused or exacerbated by polar exploration. This narrative review examines the spectrum of cutaneous manifestations observed in polar explorers and researchers, providing insights into both prevention and management of diseases in these extreme environments. Considering the increasing relevance for polar research due to changes to the poles from climate change and its relevance as a space analog for both human space exploration and extremophile study for astrobiology research, understanding and anticipating the health challenges of polar inhabitants in paramount especially considering the logistical challenges of delivering healthcare in this environment. The notable risks posed to skin health demonstrate the important role dermatologists can play in developing preventative guidelines, treatment recommendations, and patient education for polar explorers, especially on how to prevent dangerous exposure to environmental hazards (Fig 1).

## Methods

We conducted a literature review using PubMed and Google Scholar between December 2024 and January 2025 to collect and analyze references that were relevant to dermatological insults and conditions present in the Arctic and Antarctic environment. Inclusion criteria included prior original research articles, literature reviews, and case reports relevant to dermatology and polar exploration that could be incorporated into the narrative structure of the review. We used Boolean operators to find literature relevant to our review using search terms, including: “hypothermia,” “gangrene,” “basal cell carcinoma,” “squamous cell carcinoma,” “melanoma,” “skin,” “dermatology,” “cutaneous,” “derm,” “Antarctica,” “arctic,” “polar,” “xerosis,” “solar,” “ozone,” “nutrition,” “polar t3 syndrome,” “vitamin,” “cold,” “dermatoses,” “overwinter,” and “healthcare delivery.” Relevant sources to the review were incorporated in a narrative bases to highlight various dermatological relevant conditions in the polar environment in terms of epidemiology, diagnosis, management, and prevention. Historical literature of polar exploration was also incorporated for illustrative examples in the introduction and discussion.

## Discussion

### Solar radiation exposure

The Ozone layer serves as an atmospheric barrier that protects against harmful ultraviolet (UV) radiation. In polar regions, particularly Antarctica, the ozone layer is significantly thinner, especially during the spring months when the ozone layer becomes depleted [15]. This thinning results in more UV-B radiation reaching the Earth’s surface, increasing exposure risks in these areas [16]. The ozone thinning over Antarctica is not only larger but also more persistent than that over the Arctic, amplifying the risks of UV-related damage in the Southern Hemisphere [17]. Contrasting this, are the much more stable values seen in Ozone measurements in San Diego and Alaska [15]. The significant spike in UV-B radiation during certain months of the year has been confirmed in several other studies [16]. One such study utilized highly sensitive *Bacillus subtilis* spores and measured UV-B radiation over several months in order to determine changes in the Ozone layer over time. This revealed that UV exposure was significantly more intense during the spring and associated with Ozone depletion or thinning [16].

Snow and ice are highly reflective substances, responsible for reflecting 80–90% of the UV radiation they receive [18]. The albedo effect describes this reflective process of light by a material. Snow and ice are known to have high albedo effects, meaning those exposed to high amounts of snow and ice, are routinely exposed to much higher amounts of radiation as well [18]. High levels of UV exposure over time significantly increases the risk of damage to the skin and eyes including conditions like photokeratitis, actinic keratoses and non-melanoma skin cancers [18]. Studies that aimed to quantify this risk showed that outdoor workers in Antarctica fitted with personal dosimeters near their faces were shown to receive up to 584.03 Standard Erythemal Doses (SEDs) cumulatively and 67.9 SEDs per day [19]. Safe standard SED exposure is recommended not to exceed 1.0-1.3 within an 8-hour period [20]. Prolonged exposure past these levels are associated with increased risk of DNA damage in skin cells and accelerated photoaging [21]. Increased exposure to UV radiation in outdoor ski workers in Spain were shown to have a higher prevalence of actinic keratoses than that of the average population [18].

There are several photodermatoses associated with UV exposure. A common condition related to sun exposure is actinic keratosis (AK), or solar keratosis. AKs are chronic and recurring in situ skin neoplasms that have a high rate of developing into squamous cell carcinoma (SCC) [22]. These lesions often appear on chronically sun-exposed skin, frequently on the face, arms, and neck [22]. Significant and long-term exposure to the sun causes pathological changes in keratinocytes, eventually leading to these abnormalities [22]. AKs appear as rough, scaly patches, diagnosed via dermoscopy and, commonly, punch biopsy [23]. Sustained sun damage after AK formation may result in the development of non-melanoma skin cancer [22]. Individuals with AKs have been shown to have a markedly higher risk (HR 5.1, 95% CI 4.7–5.6) of developing any skin cancer and an even higher chance (HR 7.7, 95% CI 6.7–8.8) of developing SCC [24].

Polymorphous light eruption (PMLE) is one of the most common forms of photodermatitis in the world [25]. This reaction occurs in patients mostly between the months of March and June each year when UV exposure is typically at its highest [25]. These pruritic skin lesions develop shortly after sun exposure and resolve with the avoidance of further exposure [25]. Topical glucocorticoids and antihistamines may aid in symptomatic relief and accelerate improvement [25]. A more severe and rare photodermatoses, known as solar urticaria, presents with urticarial lesions shortly after excessive sun exposure. If enough skin is overexposed to UV radiation, anaphylactic shock may occur. Due to the severity of solar urticaria, topical steroids are not sufficient, and patients must undergo photochemotherapy to prevent repeated anaphylaxis [25].

Typical sunburns are acute inflammatory reactions occurring due to extended exposure to UV light [26]. These burns can range in severity, and repeated occurrences are correlated with an increased risk of skin cancer development [26]. A city in Chile, affected annually by polar ozone thinning, has been shown to have a higher incidence of sunburns due to an increase of 300 nm in radiation [17]. Both increased ozone thinning, combined with the reflectivity of snow, contribute to the increased dermatological risks experienced by polar explorers and researchers. Individuals in these areas must take extra care to prevent such conditions due to unintended excessive UV exposure. Sun-protective clothing, hats, sunscreen, and snow goggles can prevent unnecessary skin exposure as well as ocular damage in these cold environments [19].

### Frostbite, hypothermia, exposure, & gangrene

Frostbite represents a significant dermatological concern in polar regions, particularly among explorers, researchers, and staff members exposed to the prolonged cold environments. Documented cases from early expeditions, such as the Franklin Expedition, demonstrate the profound impacts of cold exposure on human tissue, with necrosis and loss of digits commonly reported [27]. Frostbite occurs when prolonged exposure to subfreezing temperatures causes ice crystals to form intracellularly and/or extracellularly within the skin and deeper tissues, leading to ischemic damage [28]. Severe frostbite can result in vesiculation, necrosis, and eventual gangrene, often requiring amputation for affected extremities [28].

Gangrene, often a secondary consequence of frostbite or other ischemic injuries, has been a historical and contemporary concern in polar regions. During the *Nimrod* expedition of 1907–1909, members reported gangrenous changes in digits due to extended exposure to cold and inadequate protective gear while climbing Mount Erebus [27]. More recently, cases among modern polar researchers have highlighted similar risks, where equipment failure or environmental hazards can lead to prolonged exposure and subsequent gangrenous complications [29]. Management often involves surgical debridement or amputation [27].

Hypothermia, while a systemic condition, also impacts skin health by reducing perfusion and delaying wound healing. Early symptoms of hypothermia include pallor and cold, clammy skin, progressing to cyanosis and gangrene as core temperature drops [30]. Instances from Antarctic research stations have reported mild to moderate hypothermia among personnel stranded during extreme weather events [31]. One account from the 1980s described two researchers rescued with hypothermia and early frostbite after being stranded onshore after their small vessel capsized [31]. Rewarming protocols, including the use of warm water immersion, were successfully employed, although one individual developed severe skin complications requiring extended treatment [31].

Cold injuries remain a notable concern in Antarctic environments, with frostbite comprising 95% of cases, followed by hypothermia (3%) and trench foot (2%) [32]. Superficial frostbite was the most common presentation, accounting for 74% of cases, with the face being the most frequently affected area (47%) [32]. Recreational activities, particularly skiing and snowmobile driving, were implicated in 78% of injuries, highlighting the risks associated with leisure pursuits in extreme conditions [32]. These data underscore the importance of preventative measures tailored to recreational exposure in mitigating cold-related dermatological injuries.

Preventative strategies are critical for mitigating these risks. Modern polar explorers benefit from advanced protective clothing, including layered systems designed to minimize heat loss while maintaining mobility [28]. Regular monitoring for early signs of frostbite or hypothermia, such as numbness, tingling, or skin discoloration, is emphasized in polar survival training [30, 31]. Protective measures like hand and foot warmers, as well as maintaining adequate hydration and caloric intake, further reduce the incidence of cold-related skin injuries [28].

Advancements in treatment protocols have significantly improved outcomes for those affected by frostbite, gangrene, or hypothermia. Early rewarming techniques, such as immersion in water maintained at 37–39 °C, have been shown to reduce tissue damage and improve recovery rates [28, 31]. Frostbite-specific therapies, including the administration of thrombolytics to enhance microvascular circulation, are becoming increasingly common [28, 33]. Continued research into the dermatological impacts of cold exposure in polar regions aims to refine both preventive and therapeutic approaches for individuals living and working in these extreme environments.

### Cold-induced dermatoses

Travel and exploration in the Arctic climate may also pose various health complications as it relates to cold related dermatoses. Given that the skin acts as the primary defense against external damage, climatic factors such as extreme cold and UV radiation may disrupt normal skin physiology and put people exposed to these conditions at risk of dermatoses. Various studies have been conducted which explore how normal skin physiology is disrupted by these extreme conditions. An observational study of researchers in an Antarctic base camp showed an initial decrease in hydration and greasiness upon arrival to the arctic climate accompanied by rougher skin surface texture, followed by gradual increase in hydration and greasiness followed by improved skin roughness on day 45 after arrival [34]. These results suggest that extreme cold climates may induce the development of dermatoses through skin barrier damage as observed by the increase in roughness upon arrival [34]. However, the data also suggests that there is a robust mechanism of cutaneous adaptation to correct for decreases in hydration and greasiness [34].

Sampling of cutaneous microorganisms in individuals on the Japanese Antarctic Research Expedition has also revealed that living conditions in these environments contribute to temporal changes in the skin Malassezia microbiota [35]. In addition to an overall increase in the level of Malassezia colonization, there was also a shift in the proportions of Malassezia globosa and M. restricta to the seborrheic dermatitis and dandruff types [35]. These findings were consistent with the hypothesis that as a result of their limited ability to bathe during their stay, colonization levels of lipophilic skin microorganisms would increase [35]. Levels of Malassezia colonization in the sampled skin folds including cheeks, anterior chest, behind the ear, and sole of feet were all increased, with the scalp experiencing the largest increase in colonization [35]. Until more practical alternatives are developed for hygiene in these extreme climates, Malassezia overgrowth and the associated seborrheic dermatitis will continue to pose a risk to Arctic travelers [35].

The Arctic environment also poses an important health challenge for travelers due to the unique epidemiology and infectious disease profile of the region. An investigation of staphylococcal epidemiology in the Australian Antarctic Research Expedition has yielded interesting results highlighting the difference in colonization of coagulase-negative and positive strands [36]. This study revealed that coagulase-negative strains survived better in the Antarctic environment than coagulase-positive strains [36]. While the naturally acquired coagulase-positive stains were unable to maintain colonization on the forearm under these extreme conditions, the coagulase-negative strains thrived during the winter [36]. These findings reinforce that a one-size-fits-all approach to healthcare for Arctic expeditioners is not appropriate [36]. For example, when dealing with treating staphylococcal infections in patients living in Arctic environments, antibiotics should be tailored to include common coagulase-negative strains, which often necessitate border-spectrum coverage [36]. More research in the field of cutaneous infections is needed to fully understand the unique risks that humans in this environment face.

### Challenges for healthcare delivery

The remoteness of polar regions makes medical providers among the most isolated in the world [37, 38]. Each station faces unique challenges due to environmental variations, but all share the necessity for high levels of self-sufficiency in medical care [38, 39]. When incidents that result in severe injury require care beyond what is available, support is needed from another facility or evacuation off the ice [39, 40]. During winter, MEDEVAC is unreliable and only feasible when the ice thaws in summer. Some regions, like the South Pole (SP) station, lack the geographical capacity for MEDEVAC [37, 39]. Additionally, sea conditions frequently hinder access to medical assistance [40]. Evacuations from expeditions present many challenges from cost to the dangers of evacuation in extreme conditions [41]. Despite extensive screening measures to minimize risk, MEDEVACs still occur across Antarctic stations [39].

Logistical barriers also complicate emergency care. Even well-equipped countries like the U.S. and Russia, face challenges in providing aid due to the vast distances and extreme conditions [37]. In certain circumstances, evacuations require coordination between treaty nations, involving multiple transfers and diplomatic negotiations [37]. Environmental factors such as blizzards and hurricane-force winds can delay transfers, while limited on-site resources demand that patients be stabilized for extended periods [37, 40]. Additional challenges include maintaining sanitation, preventing carbon monoxide poisoning from poorly ventilated heating, and addressing dermatological issues caused by cold, dry conditions [41]. Climate change further exacerbates these challenges, contributing to the rise in vector borne diseases [38].

Unexpected medical emergencies and injuries can still occur, even among healthy, screened expeditioners [37, 40]. In remote settings, medical practitioners must be prepared to manage a variety of conditions with limited resources. It is important that pharmaceutical stocks account for long-term care needs, and medications must remain stable in extreme environments [41]. Common conditions are allergic reactions, frostbite and sunburn which due to the isolation and extreme cold are associated with heightened risks of deteriorating [39, 41]. Providers must balance the risks of rushed procedures against environmental conditions to avoid preventable casualties [41]. A general concern with conditions in polar regions is lack of reporting which causes the true prevalence of them to be unknown [41].

Telemedicine has become essential in polar healthcare, allowing first responders to receive remote consults from distant medical providers with reduced logistical burdens. Modern technology now enables consultations from a distance through the use of audio and video, improving diagnostics in specialties like dermatology. However, extreme weather and bandwidth limitations still challenge communication systems [40]. Emerging technologies, such as augmented reality (AR) and robotics, offer potential solutions by enhancing remote surgical guidance and simulation-based training [38, 40]. The use of artificial intelligence and drones may help with diagnosis, supply delivery, and search-and-rescue missions, though they raise ethical and security concerns [38]. These advancements must prioritize patient privacy and adhere to regulations like HIPAA [38].

Physicians in polar regions must be highly skilled and adaptable, capable of improvising with limited resources [37]. Recruiting medical providers poses a challenge in itself as it requires an individual with skills to deal with the demands of remote practice, isolation from society, length of time spent away and for Australian wintering expeditioners– having a prophylactic appendectomy [37]. Availability of providers with years of medical experience along with some surgical experience to accommodate emergencies can be a difficult find. Providers often work alone, which requires other personnel to assist with basic medical tasks when needed [40]. Amongst the unpredictability and stress dealing with polar weather, providers must maintain the same level of care and confidentiality as in conventional settings [38, 41]. Expedition conditions are stressful, placing providers at risk while also being responsible for the wellbeing of others [41]. Due to the very strict screening process, providers must be weary that patients could not be disclosing their complete medical history [41]. There is need for improvement in screening criteria as individuals who are deemed fit to travel to the polar regions may still be placing a disproportionate burden on resources [39].

### Dermatologically relevant hormonal and nutritional changes

The extreme cold and low-light environment of the polar environment has been linked to hormonal dysregulation (Polar T3 Syndrome) and nutrient deficiencies, especially in winter months [42, 43]. Additionally, the remote environment of poles makes delivery of fresh foods difficult, and delivery ceases during overwintering. The changes in circadian rhythm, light exposures, and metabolic demands due to exposure to extreme colds in overwintering researchers and explorers are believed to be the cause of changes in triiodothyronine kinetics in a condition known as Polar T3 Syndrome, which presents with cognitive and mood disorders [44]. Although not reported in Polar T3 studies, deficiencies in triiodothyronine and hypothyroidism lead to cutaneous changes, such as myxedema, hair embrittlement, alopecia, nail embrittlement/thinning, epidermal thinning, and xerosis [45]. Investigation into the potential synergy of low triiodothyronine coupled with the environmental effects on the skin directly could be investigated. The reliance on canned food and increased metabolism to maintain core body temperatures may lead to increased metabolism of nutrients (particularly water-soluble vitamins) [42, 43]. Vats et al. in a 2007 study of members of an Indian Antarctic expedition found statistically significant decreases in Vitamin C (ascorbic acid) levels among the participants after one month in the Antarctic, however the values were still within the normal reference range [46]. Considering the adverse sequela of ascorbic acid deficiency mentioned above and the role ascorbic acid can serve in helping to regulate body temperature, Reynolds et al. recommend supplementation for polar explorers of 250 mg daily [47]. Cabalin et al. in a meta-analysis of Antarctic expeditions reported Vitamin D deficiency (correlated with non-scarring alopecia in other studies) in Antarctic explorers with notable prevalence in overwintering expeditions and reported improved Vitamin D status with supplementation recommending explorers use Vitamin D supplementation to prevent deficiencies [43, 48]. Clinically significant vitamin B3 (niacin) deficiency implicated in the dermatologically relevant condition of pellagra is postulated to be uncommon in polar explorers due to biosynthesis from tryptophan, thus Reynolds et al. recommend a target niacin intake of 20 mg daily to avoid niacin toxicity, while ensuring adequate intake [47].

## Conclusion

The extreme environments encountered in the polar regions contribute to various dermatologic complications, including frostbite, hypothermia, and dermatoses. These complications are further exacerbated by nutritional deficiency, hormonal dysregulation, increased UV radiation exposure, and limited access to rapid, comprehensive medical care. Without proper preventative and management strategies, cutaneous disorders can compromise the health of polar explorers and hinder the success of expeditions. Several strategies include the use of protective apparel to mitigate frigid temperatures and protect personnel from UV-induced dermatoses, rewarming techniques such as warm water immersion to reverse frostbite and hypothermia progression, and vitamin supplementation to counter nutritional deficiencies contributing to dermatologic complications. Proper hygiene is another essential area for preventing seborrheic dermatitis and preserving skin-barrier integrity. Telemedicine has helped bridge the gap in accessing rapid, medical expertise, however, access to comprehensive medical care remains a challenge in emergency settings. On-site artificial intelligence, augmented reality, and drone technology present as possible solutions to delivering urgent care. Additionally, further research into the dermatologic manifestations of Polar T3 syndrome and its treatment options would be valuable in ensuring the safety and success of future polar expeditions. As polar research grows, dermatologists can serve a crucial role by developing protocols for managing dermatologic complications, training healthcare personnel prior to polar missions, and providing remote mission support through telemedicine.

## Data Availability

No datasets were generated or analysed during the current study.
